# Assessment and Characterization of Some New Photosensitizers for Antimicrobial Photodynamic Therapy (aPDT)

**DOI:** 10.3390/ma13133012

**Published:** 2020-07-06

**Authors:** Laura Monica Dascalu (Rusu), Marioara Moldovan, Doina Prodan, Irina Ciotlaus, Violeta Popescu, Ioana Baldea, Rahela Carpa, Sorina Sava, Radu Chifor, Mindra Eugenia Badea

**Affiliations:** 1 Department of Prosthodontics and Dental Materials, Iuliu Hatieganu University of Medicine and Pharmacy, 31 Avram Iancu Str, 400083 Cluj-Napoca, Romania; rusu.monica.laura@gmail.com (L.M.D.); savasorina@yahoo.com (S.S.); 2 Institute of Chemistry Raluca Ripan, Babes-Bolyai University, 30 Fantanele Str, 400294 Cluj-Napoca, Romania; doina.prodan@ubbcluj.ro (D.P.); irina_ciotlaus@yahoo.com (I.C.); 3 Physics and Chemistry Department, Technical University of Cluj-Napoca, 28 Memorandumului Str, 400114 Cluj-Napoca, Romania; violetap2003@gmail.com; 4 Department of Physiology, Iuliu Hatieganu University of Medicine and Pharmacy, 400083 Cluj-Napoca, Romania; baldeaioana@gmail.com; 5 Department of Molecular Biology and Biotechnology, Faculty of Biology and Geology, Babeș Bolyai University, 1 M. Kogălniceanu Str, 400084 Cluj-Napoca, Romania; k_hella@yahoo.com; 6 Department of Preventive Dental Medicine, Iuliu Hatieganu University of Medicine and Pharmacy, 31 Avram Iancu Str, 400083 Cluj-Napoca, Romania; raduchifor@yahoo.com (R.C.); mindrabadea@gmail.com (M.E.B.)

**Keywords:** photosensitizers, gels, biofilms, antimicrobial photodynamic therapy

## Abstract

The novelty of this study consists on the formulation and evaluation of five complex experimental natural photosensitizers (PS): gel with oregano essential oil (O), gel with methylene blue (AM), gel with a mixture of essential oils (Thieves-H), gel with arnica oil and curcuma extract (CU) and gel with frankincense essential oil (T), used as photosensitizing agents (PS) in antimicrobial photodynamic therapy (aPDT) in the control of microbial biofilm in oral cavity. The experimental PS were characterized by gas chromatography-mass spectrometry (GC-MS), Fourier-transform infrared spectroscopy (FTIR), UV-Vis spectroscopy, cytotoxicity assay, antimicrobial effect and scanning electron microscopy (SEM). The IR spectra of the experimental PS with essential oils exhibit absorption bands due to the presence of water and glycerol in high quantities. The studied compounds had a reduced cytotoxic effect on cell cultures. The lowest cytotoxic effect was observed in experimental PS with oregano essential oil and methylene blue PS. Essential oils with proven antibacterial capabilities used in experimental PS confer antibacterial activity to the gels in which they are incorporated, an activity that may be more efficient use of a PDT therapy. Single bacteria were detected mainly by SEM after 12 h, while aggregate bacteria and micro colonies dominated the samples at 48 h.

## 1. Introduction

Bacteria that grow in biofilms adhere to the surface of the tooth, where they multiply and form micro colonies embedded in an extracellular polymeric matrix, which includes water and nutrient channels. Biofilms that colonize dental surfaces are among the most diverse and complex biofilms that exist in Nature. There are more than 700 bacterial species that can lead to periodontal disease. Currently, the most common method of treating periodontal disease is the mechanical removal of periodontal biofilms. Antimicrobial agents are also used for periodontal treatments, but biofilm species have developed several mechanisms of resistance to this. Removal of oral microflora and the struggle to maintain antimicrobial agents concentrated in the oral cavity leads to problems associated with the use of these antimicrobial agents [1,2,3,4].

Photodynamic therapy (PDT) is a non-invasive therapeutic alternative used to treat bacterial, fungal and viral infections, minimizing the occurrence of bacterial resistance and can be considered an adjuvant to conventional mechanical therapy. Therefore, antimicrobial PDT (aPDT) may be an interesting therapeutic approach in periodontal and endodontic therapy and may be considered as a substitute for chemotherapy. aPDT is currently available for the treatment of periodontal, peri-implant disorders and endodontic therapy [5]. 

Sometimes in severe cases of periodontitis, adjuvant use of antibiotics cannot cope with resistant bacterial strains, therefore alternative treatment modalities such as aPDTare needed [6,7]. 

PDT is based on an oxygen-dependent photochemical reaction, which involves the activation of a photosensitizer (PS) in the presence of a harmless visible light source, generating reactive oxygen species, especially simple oxygen, which induces injury and death of microorganisms, without affecting the host cells [8,9,10,11]. Photosensitizers are molecules able to absorb light at a specific wavelength, leading to photochemical or photo physical reactions [12].

The selection of PS used for PDT in dentistry depends on the used light source. The most common PS used are haematoporphyrin (620–650 nm), phenothiazine (620–700 nm), cyanine (600–805 nm), phototherapeutic agents (550–700 nm), phthalocyanines (660–700 nm) and chlorines. The dyes most studied and used in PDT are phenothiazines in several concentrations [13,14,15,16,17].

According to the literature, three light sources are used in PDT: laser, light emitting diodes (LED) and halogen lamps [18,19].

According to specialists, the ideal PS associated with a light source should not be cytotoxic or have low toxicity to the host cells but should have instead high toxicity to the target cells [20,21]. 

Several studies have shown that oral bacteria are sensitive to PDT in planktonic cultures. Recent studies have reported that PDT induced bacterial cell destruction and reduced bacterial counts by more than 10-fold in biofilms of *Streptococcus mutants* [22,23,24,25,26], *Streptococcus sobrinus* and *Streptococcus sanguinis*, using toluidine blue or erythrosine as a photosensitizer [2,27,28], silver nanoclusters/rose Bengal nanocomposite [22], curcumin and methylene blue [24].

The novelty of the study consists on the formulation and evaluation of five complex, experimental photosensitizers: gel with oregano essential oil (O), gel with methylene blue (AM), gel with a mixture of essential oils (Thieves-H) which contains clove, cinnamon bark, lemon, *Eucalyptus radiata* and rosemary essential oils, gel with arnica oil and curcuma extract (CU) and gel with frankincense (T) essential oil. The experimental gels are used as photosensitizing agents in antimicrobial photodynamic therapy for the control of oral cavity microbial biofilms. The cytotoxicity study was performed on oral dysplastic keratinocytes (DOK, ECCAC 94122104, Sigma Aldrich, Heidelberg, Germany), for the control sample and experimental gels with essential oils, methylene blue and curcuma. We aimed to identify and quantify the antibacterial effect of the natural experimental photosensitizers and control sample, on examples of two classes of bacteria: Gram positive (*Staphylococcus aureus* ATCC 25923) and Gram negative (*Escherichia coli* ATCC 25922).

It was chosen to test and quantify their antibacterial effect by using a qualitative diffusimetric method which is simple, fast and easy to use. This method has the advantage that it can be used for almost all antimicrobial substances. It was also desired to test the efficiency of photodynamic antimicrobial therapy on extracted teeth by scanning electron microscopy. As far as we know there is only one study that demonstrates the efficiency of photodynamic therapy using essential oils of eucalyptus (*Eucalyptus radiata*), clove (*Eugenia caryophyllata*) and thyme (*Thymus vulgaris*) on *Staphylococcus epidermidis* (CECT 231), *Pseudomonas aeruginosa* (CECT 110), and *Candida albicans* (1392) [25]. Marqués-Calvo et al. [25] concluded that the photodynamic activity depends on the oil concentration and light source (red or blue light) and that phenols (eugenol and thymol) were the most potent antimicrobial agents, underlining that light exposure improved antimicrobial activity. Our study is the first study that approaches the investigation of essential oils (arnica oil from the sample containing curcuma, oregano, frankincense and Thieves blend) as photosensitizers for *Escherichia coli* and *Staphylococcus aureus.*

## 2. Materials and Methods 

### 2.1. Materials

#### 2.1.1. Experimental Photosensitizers (PS)

The experimental studies were performed using gels based on natural compounds and methylene blue as photosensitizers in PDT. For PS formulation, we used three essential oils based on frankincense (T, *Boswelia carteri*), oregano (O, *Origanum vulgare*), Thieves blend (H), one with methylene blue (AM) and one (CU) with curcuma (*Curcuma longa*) and arnica oil; gelatin, (GE-99.5%), glycerin (GY), Kaqun^®^ water (K) and salicylic acid (AC-99%).

Experimental PS contain nanocapsules, which include an organic phase based on essential oils, with the active principle wrapped in a fine film of polycaprolactone (PLC) to ensure controlled release of the active substance through the diffusion phenomenon. Nanocapsules were prepared by mixing essential oil and PCL (Sigma-Aldrich Inc., St. Louis, MO, USA) with polyvinyl alcohol (Sigma-Aldrich Inc.) as the surfactant and ethyl acetate, (Sigma-Aldrich Inc.) all of them in varying concentrations (1, 2.5 and 2%).

The gels were prepared from a mixture of gelatin: glycerol (Sigma-Aldrich Inc.) in a weight ratio of 1:1 and 60 mL Kaqun^®^ water (Harghita, Romania), using the following procedure: gelatin and glycerol with 0.015% salicylic acid solution were added to the water. The gels formed were divided into five equal parts: in three parts it was added essential oil (T, H, O—Young Living, Groningen, The Netherlands), in one part it was added curcuma extract with arnica oil (Plant extract, Cluj-Napoca, Romania) and in one part it was added methylene blue (Sigma-Aldrich Inc.). Curcuma extract was made from fresh turmeric root.

#### 2.1.2. Photodynamic Therapy Protocol (PDT)

In order to evaluate the efficiency of photodynamic therapy of the formulated experimental PS, a protocol on extracted teeth was developed. The study used 30 extracted teeth with no enamel lesions (*n* = 30). The extracted teeth were immersed in natural saliva, collected from subjects with high carioreceptivity, and then incubated at 37 °C at least 48 h. After the initial examination, PS was applied on the teeth surface for 4 min. After this time, PS was removed by rinsing with 0.9% saline, taking care that the jet did not act directly on the area of interest to avoid mechanical removal of bacteria. Dental surfaces were subjected to photodynamic therapy using the HELBO TheraLite Laser (Helbo Photodynamic Systems GmbH & Co., Wels, Oberösterreich, Austria) for 1 min. This diode device has a 660 nm wavelength and 40 mW/cm^2^ power range. To evaluate the antibacterial effect, dental surface was examined by SEM before and after application of photosensitizers and laser irradiation. The research was performed on extracted teeth and all subjects gave their informed consent for inclusion before they participated in the study. The study was made according with the Declaration of Helsinki, and the protocol was approved by the Research Ethics Committee of the University of Medicine and Pharmacy “Iuliu Hatieganu”, Cluj-Napoca, Romania (authorization no. 578/10.12.2019).

### 2.2. The Gas Chromatography-Mass Spectrometry (GC-MS)

For this study, four types of gels were used as antibacterial agents with applications in dentistry: gel with arnica oil and curcuma extract (CU), gel with oregano essential oil (O), gel with frankincense essential oil (T) and gel with Thieves (H), a mixture of essential oils. GC-MS analysis was used to identify the chemical composition of tested essential oils. For this gel (0.5 g) was dispersed in hexane (10 mL, VWR International, Fontenay-sous-Bois, France) for 2 h, then ultra-sonicated for 15 min and then centrifuged at 4400 rpm for 15 min. The volatile-containing hexane fraction was filtered and then dried over sodium sulfate. The essential oil recovered from gel was injected into the GC-MS.

An Agilent GC-MS Gas Chromatograph-7890A/5975/2008 (Agilent Technologies, Inc. Europe, Waldbronn, Germany) was used for analysis; GC-MS analyses were performed in scan mode on a DB-5MS (30 m × 0.25 mm × 0.25 µm) capillary column (Agilent 19091S-433M), high purity He carrier gas at a flow rate of 1 mL/min. Temperature program: initial temperature 40 °C with a ramp rate of 8 °C/min up to 220 °C, then with 20 °C up to 280 °C and maintained 5 min, injector temperature 250 °C, injection volume of 1 µL, 100:1 slide, MS 70 eV, mass range u.a.m. 30–400. NIST library was used for identification/confirmation of the components structure. In addition, a C8-C20 standard alkanes solution (Alkane Standard Solution C_8_-C_20_, Sigma Aldrich) was used for calculation of the linear retention index (RI) and matching the experimental values with those reported in the literature for similar chromatographic columns, in the same conditions.

### 2.3. Characterization of Experimental PS by UV-VIS Spectroscopy

UV-VIS spectra of the gels have been recorded using a Lambda 35 UV-VIS spectrometer (Perkin Elmer, Waltham, MA, USA). Because the absorption peaks of the gels were very strong, in order to obtain better UV VIS absorption spectra, we recorded the spectra using experimental PS samples diluted with water (for samples AM, H, T) or dimethyl sulfoxide (DMSO, for sample CU).

### 2.4. Determination of IR Spectra for Experimental PS (Essential Oil Gels)

In order to record the FTIR spectra of gels and samples of dried gels in an oven, up to constant weight at 30 °C, a Spectrum BX FTIR spectrometer (PerkinElmer, Sunnyvale, CA, USA) provided with an Attenuated Total Reflectance (ATR PIKE MIRacle^TM^, Madison, WI, USA) with a diamond crystal plate was used. The resolution was 2 cm^−1^ in the range of 4000–800 cm^−1^.

### 2.5. Cytotoxicity Determination of Experimental Gels

#### 2.5.1. Cell Culture

Evaluation was performed on dysplastic oral keratinocytes (DOK, ECCAC 94122104, Sigma Aldrich, Heidelberg, Germany) used at their 31st–32nd passage. DOK were cultured in Dulbecco’s modified Eagle’s medium (DMEM) supplemented with 5% fetal calf serum, 5 μg/mL hydrocortisone, 50 μg/mL gentamicin and 5 μg/mL amphotericin (Biochrom Ag, Berlin, Germany) in standard cell culture conditions (37 °C, 60% humidity and 5% CO_2_); medium was changed twice a week.

#### 2.5.2. Extract Preparation

The culture medium conditioned with the photosensitizer was obtained according to the method described by Cavalcanti et al. [29] in compliance with the ISO 10993–12:2012 [30] procedures. Conditioning was performed at room temperature for 30 min using 0.2 g of each tested gel/mL of culture medium and immediately after, the conditioned medium was diluted to 0.001, 0.002, 0.005, and 0.01 in medium and applied to cell cultures.

#### 2.5.3. Cytotoxicity Assay

The cytotoxicity assay aimed to study the possible toxic effects of the experimental gels exposure against keratinocytes of the oral mucosa, in order to assess the biocompatibility of these compounds. Therefore, an MTS assay (a variant of MTT test) using a CellTiter 96^®^AQueous Non-Radioactive Cell Proliferation Assay (Promega Corporation, Madison, WI, USA) was employed to assess cell viability. DOK cells were grown at a density of 10^4^/well in 96 well flat bottom plates (TPP, Trasatingen, Switzerland) and allowed to settle for 24 h. Then the cells were exposed to the extract of each photosensitizer, prepared as described above, in dilutions of: 0.001, 0.002, 0.005, and 0.01 for 24 h. The cells were then washed and the viability was immediately measured by colorimetric measurement of a dye compound-formazan, generated by metabolically active, viable cells. Measurement was done using an ELISA plate reader (Tecan, Männedorf, Switzerland) at 540 nm. All experiments were performed in triplicate. Untreated cell cultures were used as controls. The results are presented as OD540. A decrease in viability below 70% of the untreated control was considered a sign of toxicity. 

#### 2.5.4. Statistical Analysis

The statistical difference between experimental materials and control groups were evaluated by two-way ANOVA and Student t-Test, followed by Bonferroni posttest; all the values in text and figures are expressed as mean ± standard deviation; results were considered significant for *p* ≤ 0.05. Statistical package used for data analysis was Prism version 4.00 for Windows (GraphPad Software, San Diego, CA, USA).

### 2.6. Determination of the Antimicrobial Effect of the Experimental Gels

The antimicrobial effect of the experimental PS (2.1.1) was tested using two sources of irradiation with LED/Laser light, compared to the control samples (CONTROL-not irradiated), on two types of bacteria: *Escherichia coli* (Gram negative) and *Staphylococcus aureus* (Gram positive). The irradiation time was 60 s. 

**Figure 1:**
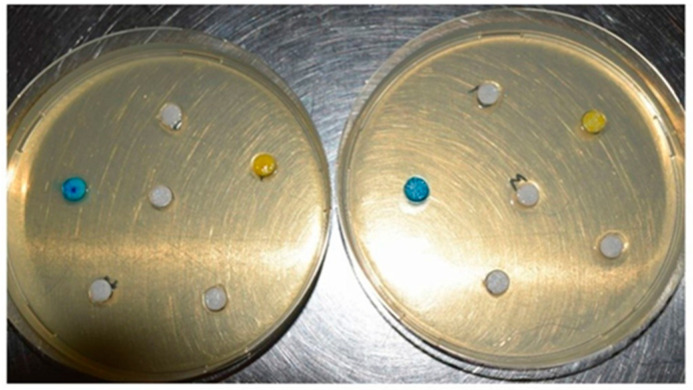
Application of various types of gels on sterile disks (left = *E. coli*; right = *S. aureus*).

The method of gel-wrapped discs (paper discs) applied to the solid culture medium is a method adapted according to the diffusimetric method of the antibiogram described by Carpa et al. [31]. Petri dishes with Mueller-Hinton culture medium were inoculated with 200 μL of test strain with a turbidity of 0.2 McFarland. After removing the excess, the plates were incubated 1 h at 37 °C. In each Petri dish, six sterile paper circles with 6 mm diameter were placed on the culture medium. On these circles, 12 μL of each gel was pipetted (Figure 1). Incubation was continued for 24 h at 37 °C and then the diameter of inhibition zone (the area in which the bacterial growth was affected by the gels impregnated into the sterile disks) has been measured. 

The size of these areas of inhibition actually indicates how we quantify antimicrobial activity.

### 2.7. The Efficiency of Photodynamic Antimicrobial Therapy on Extracted Teeth

#### Microstructural Analysis by SEM

SEM was carried out on the surfaces of uncoated samples attached to carbon tabs on a Scios field emission scanning electron microscope (FEI, Inspect S, Hillsboro, OR, USA), operated at an accelerating voltage of 1.0 kV. After the teeth were incubated in the saliva, they were allowed to dry and then examined by SEM. The effect of antimicrobial photodynamic therapy on tooth enamel biofilm was evaluated compared to a commercial material-HELBO TheraLite (Helbo Photo-dynamic Systems GmbH & Co, Wels, Oberösterreich, Austria). At the initial examinations, we aimed to characterize the oral biofilm.

## 3. Results

### 3.1. GC-MS Analysis of Gels Containing Essential Oils

GC-MS analysis shows the volatile compounds specific to experimental PS: arnica oil (A) from the sample containing Curcuma, oregano essential oil (O), frankincense essential oil (T) and Thieves essential oil (H). The volatile compounds recovered from the composition are listed in Table 1.

The GC-MS analysis shows the specific volatile compounds found in the sample H containing, as shown in Table 1, a blend of essential oils. Clove essential oil (*Syzygium aromaticum*) is a mixture of different constituents, having two main active ingredients that are eugenol and caryophyllene. They contribute to the antimicrobial and antioxidant properties of the essential oil. The main compounds were limonene, γ-terpinene, β-pinene, myrcene, sabinene and citral (Table 1). The frankincense oil was characterized by the high content of the monoterpenes, which constituted 84.86% of the toal and among which α-pinene and limonene were the major constituents. The remaining 14.53% was accounted for by sesquiterpenes among which *E*-caryophyllene was the major constituent. Oregano oil has a complex composition with the main compounds being carvacrol, *p*-cymene and γ-terpinene, while arnica oil has a rich content of (*Z*)-γ-atlantone (19.95%), β–turmerone (14.24%), aR-turmerone (12.34%), and α-pinene (11.93%).

### 3.2. Determination of UV-Vis Spectra for Experimental PS 

**Figure 2:**
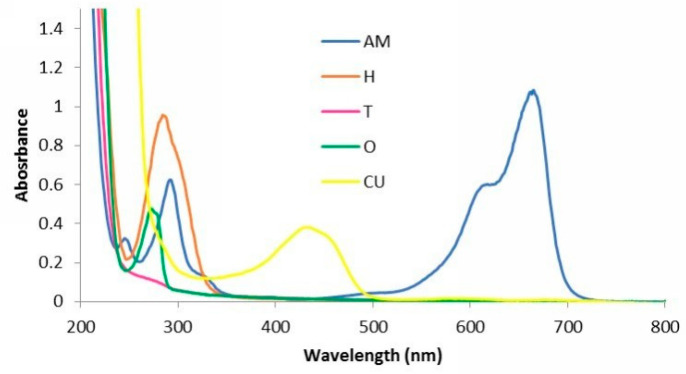
UV-VIS spectra of the experimental PS with AM, H, T, O and CU.

The UV-VIS absorption spectra are presented in Figure 2. 

The main absorption bands in the spectra of the gels appear due to the presence of essential oils, curcuma and methylene blue. In Figure 2, it could be observed that gel AM exhibited 4 absorption peaks, at 236, 289, 612 and 667 nm due to the presence of methylene blue.

Sample H containing Thieves blend has an absorption band at 283 nm, while sample O with oregano oil has a strong absorption peak at 271 nm. Samples AM, H, O and CU have a strong absorption for wavelengths smaller than 250 nm. The gel CU exhibit a large band between 300 and 500 nm, with a peak at 437 nm, due to the presence of curcuma, and a strong absorption for wavelengths smaller than 300 nm, probable due to arnica oil. According to Park and Dovigo [32,33] the absorption range of curcuma is between 300–500 nm, with maximum absorption at 430 nm.

### 3.3. Determination of IR Spectra for Experimental PS 

**Figure 3:**
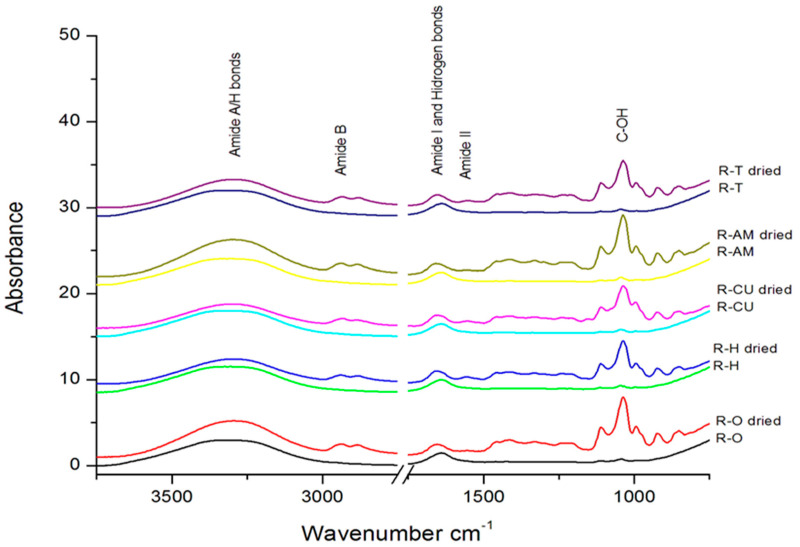
IR spectra for the experimental PS gels with essential oils and for dried gels.

The IR spectra (Figure 3) of the five PS with essential oils have similar profiles and exhibit absorption bands due to the presence of water and glycerol in high quantities. The integration of the essential oil into the polymer matrix conducted to some chemical interactions with the gelatin. In order to better understand the interaction between gelatin, glycerol and essential oils we obtained dried gels samples that have been also analyzed. In Table 2 are presented the main vibration bands from gels and dried gels (Figure 3).

### 3.4. Cytotoxicity Assessment of Natural Photosensitizers

**Figure 4:**
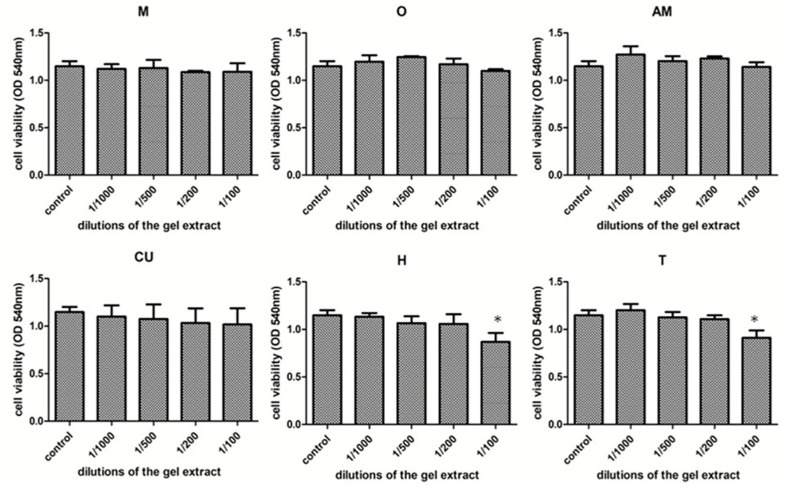
Cytotoxicity assay of the tested photosensitizers in different gel extract concentrations—0.001, 0.002, 0.005, 0.01; control PS (M), O with oregano essential oil, AM with Methylene Blue, CU with curcuma extract, H with Thieves mix essential oil, T with frankincense essential oil; Each bar represents mean ± standard deviation (*n* = 3), * represents *p* < 0.05, compared to untreated control.

The cytotoxicity profiles of the control (M) and the five experimental PS (gels with essential oils and methylene blue) are presented in different dilutions. All experiments were performed in triplicate and the results are shown in Figure 4. There were no statistically significant differences between the viability of the control group and the O-, AM-, CU-treated groups. 

However, in the case of H and T groups, there was a significant decrease in viability at the concentration of 1/100, while the other concentrations had a non-significant impact on cell viability. Two-way ANOVA test showed significant interaction between the treated groups (*p* = 0.0409). There was a statistically significant difference between the types of gel used as compared to the control group (*p* < 0.0001) and respectively to the M group (*p* < 0.0001). Viability decrease, while the concentrations used of had a lower, but still significant impact (*p* < 0.02). However, the maximum viability decrease in the case of the gel H (75.7% of the untreated control) and respectively in the case of gel T (79.4% of untreated control) is above 70% of the untreated control, considered as a toxicity limit for the viability test. Therefore, the viability results show that the experimental gels were well tolerated by the dysplastic oral keratinocytes, without signs of toxicity for any of the tested materials. 

### 3.5. The Efficiency of Antimicrobial Activity of Experimental PS

**Figure 5:**
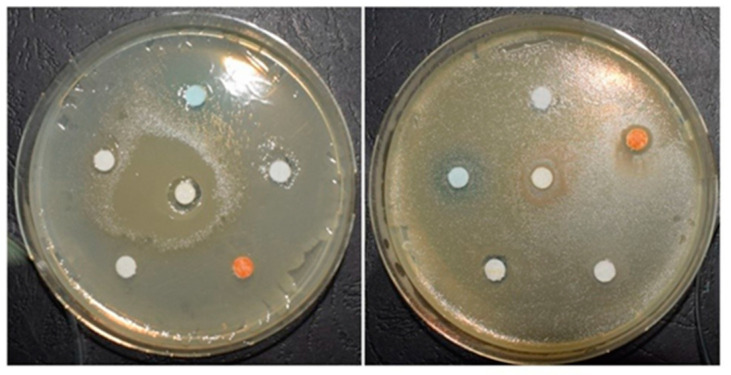
Antimicrobial activity of experimental PS after incubation (left = *E. coli*; right = *S. aureus*).

It was observed that after 24 h of incubation, areas of inhibition appeared for both Gram positive (G+) and Gram negative (G−) strains, but not for all tested samples. The diameter of the inhibition zones was relatively small, as can be seen in Figure 5.

**Figure 6:**
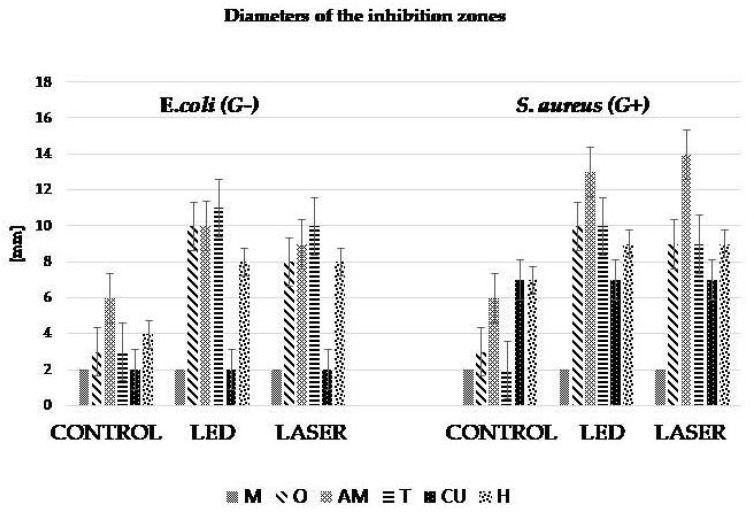
Antimicrobial activity of experimental PS—the diameters (mm) of the inhibition zone.

The diameters of the inhibition zones (mm) for *S. aureus* and *E. coli* can be seen in Figure 6.

In the absence of irradiation, for control samples, a reduced inhibition zone was found in both bacterial strains. In the presence of phototherapy, it was found that sample AM recorded the highest inhibitory value for the bacterial strain G+, in both irradiation variants (LED, Laser). For sample O, comparable values of the diameter of bacterial inhibition were obtained, on both bacterial strains, in both irradiation variants. In the case of sample T, the diameter of the bacterial inhibition zone is slightly increased for *E. coli*, compared to *S. aureus*. In contrast, in the case of sample H the diameter of the bacterial inhibition zone is slightly increased for *S. aureus*. The largest difference between the diameters of the bacterial inhibition zone was recorded in the case of sample CU-A (7 mm for *S. aureus* and 2 mm for *E. coli*), in all 3 experimental variants (CONTROL, LED and LASER). Sample CU-A recorded the same values of bacterial inhibition in the case of the bacterial strain G+, for all tested samples (Control, LED, Laser).

### 3.6. The Efficacy of Photodynamic Antimicrobial Therapy on Extracted Teeth, without Enamel Lesions. Microstructural Analysis by SEM, after Bacterial Colonization and after Treatment with PS

**Figure 7:**
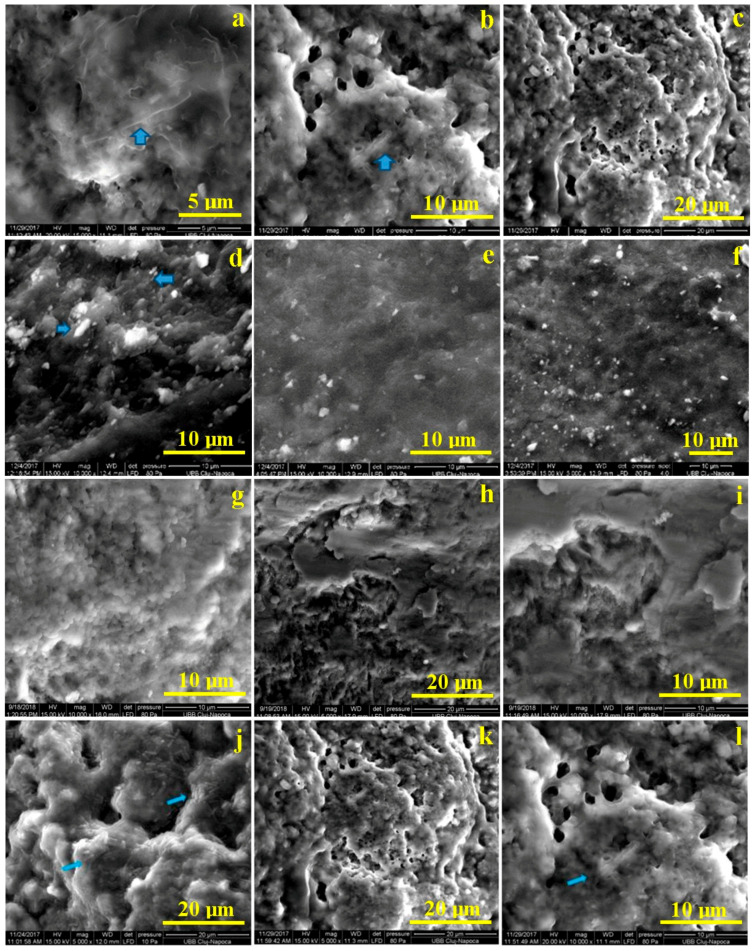
SEM images of teeth before and after treatment with photosensitizers: (**a**) after bacterial colonization, 15,000× magnification; (**b**) after treatment with commercial gel HELBO, 10000× magnification; (**c**) 5000× magnification; (**d**) after bacterial colonization, 10,000× magnification; (**e**) after treatment with experimental gel AM, 10000× magnification; (**f**) 5000× magnification; (**g**) after bacterial colonization, 10,000× magnification; (**h**) after treatment with experimental gel O, 5000× magnification; (**i**) 10,000× magnification; (**j**) after bacterial colonization, with 5000× magnification; (**k**) after treatment with experimental gel CU, 5000× magnification; (**l**) after treatment with gel CU, 10,000× magnification.

Representative images of the samples after bacterial colonization and after treatment with experimental PS and HELBO commercial PS are shown in Figure 7.

SEM images of teeth show an intact well-defined bacterial wall, without discontinuities, after bacterial colonization, (Figure 7a,d,g,j), while after treatment with commercial gel, HELBO, the arrow from Figure 7b,c shows the interrupted bacterial wall, both longitudinally and transversely. Figure 7e,f reveal that after treatment with experimental gel AM, a smaller amount of dental biofilm can be observed and the bacteria are smaller. After treatment with experimental gel CU, the arrow in Figure 7k,l also shows the interrupted bacterial wall both longitudinally and transversely. The same result (a smaller amount of bacteria) can be observed in the case of treatment with experimental gel O (Figure 7h,i).

## 4. Discussion

The present study set out to evaluating the efficiency of experimental photosensitizers with essential oils, curcumin and methylene blue when bacteria populations were illuminated with LED and laser light. The activity of essential oils is related to the respective composition, the structural configuration of volatile oils and their functional groups, and the possible synergistic interactions between components [34].

The antimicrobial activity results from the complex interaction between the different classes of compounds such as phenols, aldehydes, ketones, alcohols, esters, ethers or hydrocarbons found in essential oils. Interactions between these components may lead to antagonistic, additive or synergistic effects. Most of the antimicrobial activity in essential oils appears to derive from oxygenated terpenoids, particularly phenolic terpenes, phenylpropanoids and alcohols. Their chemical structure and charge makes their action in the photodynamic therapy process possible. According to several published reports the frankincense essential oil shows antibacterial and antifungal activity [7,8]. This herbal oil with anti-inflammatory effect may be applied in the treatment of gingivitis, which is an inflammatory disease of periodontal tissue [9,10]. The chemical structure of the phenols would allow their good absorption by bacteria and the wavelength of light would provide sufficient radiant intensities and duration to initiate the inactivation of the microorganisms.

Due to the large content of water, the FT-IR spectra of the gels revealed mainly the vibration of water and amide bands, because the vibrations for amide A and amide I bands are located into the same wavenumber ranges. At 3305 cm^−1^ for gels AM and O, 3306 cm^−1^ for gels CU and T, and 3332 cm^−1^ for gel H can be observed the vibrations of OH from free water and glycerol together with the amide A band due to NH stretching coupled with hydrogen bonds in the spectra of gels, an important red shift from 3404 cm^−1^ the absorption band of liquid water [35,36] due to symmetric stretch (ν1), asymmetric stretch (ν3) and bending (ν2) vibrations of O–H. 

In the case of dried samples, the band from around 3306 cm^−1^ suffered a small red shift, to 3296 cm^−1^ compared to gels for all samples, because in this case, the main impact on the band vibration is due to amide A vibrations, with a smaller contribution of water. The main vibration bands for gels and dried samples are presented in Table 2.

The amide B vibrations due to CH_2_ asymmetrical stretching are not visible in the gel spectra, but appear in the spectra of dried ones, at 2938 cm^−1^ for sample AM and T, 2933 cm^−1^ for CU, 2937 cm^−1^ for both dried samples H and O. At smaller wavenumbers, around 2880 cm^−1^, one can notice the absorption bands of CH_3_-symmetric stretch from proteins, only in the spectra of dried samples. 

An asymmetrical absorption band can be observed in all IR spectra at 1638 cm^−1^ for gels with essential oils and curcuma extract. This band can be assigned to the amide I band due to C=O stretching/hydrogen bonding coupled with COO– from gelatin [37] and band vibrations (ν2) of O–H bonds from water and glycerol [35]. The vibration shifts from 1643 cm^−1^ corresponding to pure water at 25 °C due to the interactions with protein molecules. 

According to Ning et al. [38] amide I absorption band for gelatin vibrates at 1658 cm^−1^. The peak of the hydrogen bonded C=O group shifts to smaller wavenumbers: 1657 cm^−1^ for sample AM and T, 1656 cm^−1^ for H, 1652 cm^−1^ for O and 1651 cm^−1^ for CU due to the disruption of C=O· · ·H–N hydrogen bonds in gelatin, and the formation of new and stronger C=O· · ·H–O hydrogen bonds between gelatin and glycerol, increasing average strength of the hydrogen bond. The amide I peak position is also influenced by the addition of essential oils and photosensitizers that have a certain influence on the formation of hydrogen bonded C=O groups as a function of their composition. 

Around 1557 cm^−1^ the amide II band due to the NH band coupled with CN stretching can be observed in the spectra of dried H and CU samples, but the absorption band is very weak in the other samples. The band that appears nearby 1414 cm^−1^ is due to the vibrations of primary –OH group of alcohol [35] in the dried samples, mainly due to the presence of glycerol. The absorption bands that can be seen around 1035 cm^−1^ in samples are due to the stretching vibration of C–OH bonds from glycerol. 

The nearly invisible peak corresponding to amide III (around 1240 cm^−1^) [39] associated with C–N stretching and N–H bending from dried samples suggests a change in secondary structure of gelatin owing to hydrogen bonding and the conformation of protein structure. The same observation regarding to the lack of amide III band in gelatin/glycerol electrospun fibers has been made by Reda Morsy et al. [39]. 

According to the literature, essential oils have antioxidant properties and can protect cells against oxidative damage. The essential oils showed different levels of cytotoxicity and also exhibited different antioxidative capacities, depending on their composition [40].

Some studies have shown that phenols (thymol, carvacrol) and monocyclic hydrocarbons (terpinolene, *R*-terpinene and γ-terpinene) are among the most active natural antioxidants found in the composition of essential oils [41,42].

Clove and cinnamon essential oils, components of the Thieves blend, were the most toxic, followed by lemon, eucalyptus and rosemary. The toxicity is most likely due to the major chemical components of the essential oils, including eugenol, limonene and 1,8-cineole [43].

Methylene blue (AM) is a phenothiazine with photosensitizing properties and antitumoral activity [24]. It has been shown that AM can induce the formation of reactive oxygen species (ROS) and other studies have correlated this with AM-induced cell death.

The investigated in vitro cytotoxicity of curcuma against primary dental pulp fibroblasts by MTS test, did not detect curcuma cytotoxicity at any of the concentrations used (25%, 50% and 100%). Moreover, the viability of primary fibroblasts of the dental pulp increased by increasing curcuma concentration. The authors [44] believe that the choice of cell line for in vitro cytotoxicity tests remains debatable, as cytotoxicity is significantly dependent on the cell line selected for the assay.

Components of natural products especially volatile terpenes and phenolic components which show antioxidant activity, can be oxidized by ROS and thus generate additional radical species like panoxyl, hydroxyl and superoxide radicals and hydrogen peroxide and affect the cellular redox status in the so-called “antioxidative stress” [45]. 

The results of the study support the idea that the viability of untreated cultures increased with exposure time, significantly for only 24 h, a marker of cell proliferation. The studied compounds had a reduced cytotoxic effect on cell cultures.

According to Hu et al. [46], photosensitizers are usually unsaturated organic molecules with double conjugated bonds, which absorb the visible spectrum, even close to the IR range and which ensure a good penetration of light into tissues. They are chosen according to the way of binding to the surface (membrane) of the microbial cells, so that aPDT can be efficient. Depending on the class of bacteria, the cell surface has a different architecture. G+ bacteria can be easily affected by neutral and anionic PS. The G− bacteria, having an additional outer membrane, with many strongly negatively charged molecules, weakly attach to a neutral PS or reject an anionic PS, reducing cell permeability. Basically, the G− bacteria, due to the cell membrane, restricts the diffusion of hydrophobic compounds through the outer membrane shell. The authors, according to data from the literature, consider that whether PS is attached to the cell surface or not, after it is photoactivated, it causes cell membrane damage [46,47,48,49,50].

In this study, we observed that in the case of *S. aureus* strain (G+), the values recorded, in terms of the area of bacterial inhibition, were higher. Also, the largest diameter of the inhibition zone is noted, in the case of gel AM. This result is due to the concordance between the wavelength corresponding to the maximum absorption of AM (667 nm), with the wavelength of the irradiation source used (LED 630 nm and laser 660 nm).

The lowest inhibitory effect was recorded in the case of gel CU. The reduced inhibitory effect was due to the fact that the absorption spectrum of gel CU (437 nm) and the wavelength of the irradiation source (LED 630 nm and laser 660 nm) were not taken into account. In the case of gels O, T and H a good inhibitory effect was obtained, regardless of the irradiation source used, for both bacterial strains.

All experimental PS had some antibacterial effect. Compared to the control sample, the antibacterial effect was amplified by irradiation with both sources (LED and laser), both for the G+ and G− strains. This result suggests that, in addition to experimental natural PS, phototherapy maximized the antimicrobial effect.

Only in the case of gel AM there was a concordance between the value of the used wavelength (LED 630 nm and laser 660 nm) and the absorption spectrum (667 nm). This concordance did not exist in the other tested PS (O 271 nm, CU 437 nm, T < 290 nm, H 283 nm). The results obtained in this study show that phototherapy acted on PS even if there was no concordance between the value of the wavelength used and the absorption spectrum of the gel.

The values obtained for gel M were insignificant for both bacterial strains and did not change after irradiation. For this sample the number of G+ and G− colonies formed was very high.

Some researchers believe that after a short treatment with aPDT, the cell surface of *E. coli* (G−) biofilms is influenced due to interference with membrane function, by inactivating membrane transport systems. Eventually, the cell surface deteriorates and although the bacteria do not die, they cannot reproduce and grow [10,20].

Mohamed et al. [51] believe that the weaker antimicrobial activity against G− bacteria compared to G+ bacteria is due to the structure of their cell walls, especially in regard to the presence of lipoproteins and lipopolysaccharides in G− bacteria that form a barrier for hydrophobic compounds. 

Oregano essential oil is a volatile aromatic essential oil whose main compounds, according to our GC-MS analysis contains are: carvacrol 71.07%, *p*-cymene 3.81%, γ-terpinene 2.13%, α-humulene 1.98%, *iso*-borneol 1.82%, borneol 1.49%, β-caryophyllene 1.27%, L-α-terpineol 1.24%, α-pinene 1.00%, terpinen-4-ol 0.95%, β-myrcene 0.74%, 1-octen-3-ol 0.63%, β-pinene 0.62%, 3-carene 0.49%, etc., in accordance with the findings of Mahomed et al. [50] who identified carvacrol, thymol, γ-terpine, p-cymene and linalool. Like the other oils, it has antimicrobial [52] and antioxidant properties [53], inhibiting the growth of *Escherichia coli* and *Staphylococcus aureus* [54,55]. Antioxidant and antibacterial properties are attributed in particular to carvacrol and thymol.

In our study we observed a sensitivity of the bacteria to this form of testing, which is, applying the photosensitizer for 4 min and then phototherapy for 1 min. Scanning Electron Microscopy allows the visualization of three-dimensional surface structures.

The results obtained from the second part of the study when enamel was used as substrate, showed again that experimental photosensitizers performed better at biofilm removing. The biofilm formed on the enamel surface, were successfully visualized after exposing the samples for 48 h to human saliva. Bacterial species were detectable on enamel samples exposed to oral fluids.

A high level of inter- and intra-individual variability was recorded. Single bacteria, as well as monolayer chains or three-dimensional aggregates of bacteria were observed, confirming the bacterial population in saliva. Single bacteria were mainly detected after 12 h, while aggregate bacteria and micro-colonies dominated the 48 h samples. Other studies that have shown incomplete eradication of oral bacteria in biofilms following the application of photodynamic therapy [56,57].

Biofilms have reduced sensitivity not only to photodynamic therapy, but also to antimicrobial treatments in general, and such disadvantages can be overcome by developing administration systems, such as nanocapsules, that significantly improve pharmacological characteristics, for example, increased local retention times, improved solubility and absorption and protection against degradation. The presence of adherent bacteria and extracellular matrix were markedly reduced and very few aggregated colonies were observed in the biofilm after photodynamic therapy.

This highlights the importance of recognizing that differently formed biofilms can provide varying results and that the type of substrate used can also determine the amount of biofilm retention.

## 5. Conclusions

Because the research in the field of dentistry is directed towards controlled release biocompatible and non-toxic transport systems, the use of cross-linking agents of natural origin is desirable. By GC-MS analysis the volatile compounds specific to the five experimental PS were determined. FTIR investigation on gel samples leads to the observations of vibration bands due to water and glycerol, while in the case of dried samples the vibrations corresponding to amide vibration bands can be observed. We also can conclude that the presence of glycerol, essential oils, curcuma and methylene blue influenced the hydrogen bonds C=O· · ·N–H from gelatin, that has been replaced by stronger C=O· · ·O–H that formed between glycerol and gelatin. The viability of untreated cultures increased with exposure time, significantly for only 24 h, a marker of cell proliferation. The studied compounds had a reduced cytotoxic effect on cell cultures. The lowest cytotoxic effect has experimental PS with oregano essential oil and methylene blue PS. By using essential oils with proven antibacterial capabilities in experimental PS, they confer antibacterial activity to the gels where they are incorporated, an activity that can be made more effective by using PDT. In the case of experimental PSAM and CU, the diameter of the bacterial inhibition zone was larger on *S. aureus* (G+) compared to *E. coli* (G−) and in the case of O the same inhibitory effect was obtained on both bacteria strains. If in the case of T the diameter of the bacterial inhibition zone is slightly increased for *E. coli*, in the case of H it is slightly increased for *S. aureus*. Single bacteria, as well as monolayer chains or three-dimensional aggregates of bacteria were observed by SEM, confirming the bacterial population in saliva. Single bacteria were mainly detected after 12 h, while aggregate bacteria and micro-colonies dominated the 48 h samples.

As far as we know our study is the first one that approaches the investigation of essential oils (arnica oil from the sample containing curcuma, oregano, frankincense and thieves mix) as photosensitizers for *Escherichia coli* and *Staphylococcus aureus*.

## Figures and Tables

**Table 1:** The volatile compounds of the essential oils recovered from the gels.

No.	Compounds	RT	RI	A%	O%	T%	H%	Family
1	α-Thujene	8.702	814	-	-	2.25	-	monoterpene
2	α-Pinene	8.865	820	11.93	1.00	42.88	2.05	monoterpene
3	Camphene	9.118	831	-	0.17	0.78	0.13	monoterpene
4	Sabinene	9.634	848	-	-	2.75	0.46	monoterpene
5	1-Octen-3-ol	9.665	849	-	0.63	-		alcohol
6	β-Pinene	9.739	852	-	0.62	1.39	3.70	monoterpene
7	3-Octanone	9.812	854	-	0.30	-		ketone
8	β-Mircene	9.908	858	-	0.74	3.56	0.46	monoterpene
9	3-Octanol	9.977	860	-	0.16	-		alcohol
10	α-Phellandrene	10.232	869	2.38	0.34	2.69	0.33	monoterpene
11	3-Carene	10.471	879	-	0.49	0.72		monoterpene
12	*p*-Cymene	10.623	883	-	3.81	2.16		monoterpene
13	Limonene	10.696	886	2.97	0.47	11.92	16.80	monoterpene
14	Eucalyptol	10.784	879	-	0.47	0.59	16.80	monoterpene
15	Gamma-terpinene	11.269	906	-	2.13	-	1.65	monoterpene
16	Linalool	11.993	930	-	0.47	0.88	2.24	monoterpene
17	α-Campholenal	12.565	949	-	-	0.46	-	monoterpene
18	*trans*-Pinocarveol	12.856	958	-	-	1.27	-	monoterpene
19	*cis*-Verbenol	12.921	961	-	-	2.09	-	monoterpene
20	Camphor	12.964	962	-	-	-	2.18	monoterpene
21	Borneol	12.973	963	-	1.49	-	-	monoterpene
22	*p*-Menta-1,5-dien-8-ol	13.202	970	-	-	0.74	-	monoterpene
23	*iso*-Borneol	13.346	975	-	1.82	-	0.46	monoterpene
24	Levomentol	13.398	976	-	-	-	0.19	monoterpene
25	Terpinen-4-ol	13.519	980	-	0.95	2.17	0.26	monoterpene
26	*p*-Cymen-8-ol	13.619	984			0.39	-	monoterpene
27	L- α-Terpineol	13.745	988	-	1.24	1.11	2.11	monoterpene
28	(-)-Myrtenol	13.861	992	-	-	0.51	-	monoterpene
29	D-Verbenone	14.117	1000	-	-	0.80	-	monoterpene
30	*cis-p*-Mentha-2,8-dien-1-ol	14.213	1003	-	-	0.46	-	monoterpene
31	Linalyl acetate	14.728	1020	-	-	0.54	-	monoterpene
32	Citral	15.028	1030	-	-	-	1.66	monoterpene
33	Cinnamaldehyde	15.127	1034	-	-	-	11.44	monoterpene
34	4-Hydroxy-3-methyl-acetophenone	15.561	1048	-	-	1.03	-	ketone
35	Copaene	16.913	1093	-	-	0.78	-	sesquiterpene
36	α-Elemene	17.130	1100	-	-	1.32	-	sesquiterpene
37	α-Caryophyllene	17.655	1119	-	-	3.60	-	sesquiterpene
38	α-Humulene	18.184	1137	0.84	1.98	0.84	-	sesquiterpene
39	2,4-Nonadienal	15.413	1043	3.47	-	-	-	aldehyde
40	Piceol	15.553	1048	2.36	-	-	-	phenol
41	Carvacrol	15.578	1049	-	71.07	-	-	monoterpene
42	2,4-Decadienal	15.795	1056	4.42	-	-	-	aldehyde
43	Eugenol	16.528	1081	-	0.38	-	35.85	monoterpene
44	β-Caryophyllene	17.664	1119	-	1.27	-	0.14	sesquiterpene
45	β-Humulene	18.188	1137	-	0.29	-	-	sesquiterpene
46	E-2-Tridecen-1-ol	18.361	1143	7.10	-	-	-	alcohol
47	Bergamotene	18.636	1153	1.70	-	-	-	sesquiterpene
48	Selina-3,7 (11)-diene	18.678	1154	-	-	0.50	-	sesquiterpene
49	Aromadendrene	18.795	1158	-	-	1.03	-	sesquiterpene
50	*trans*-Cubenol	19.064	1168	-	-	1.20	-	sesquiterpene
51	β- Farnesene	19.086	1168	1.88	-	-	-	sesquiterpene
52	Cadina-1(6),4-diene	19.129	1170	-		0.89	-	sesquiterpene
53	Caryophyllene oxide	20.126	1205	-	0.45	2.40	-	sesquiterpene
54	(-)-Globulol	20.243	1209	-	-	0.79	-	sesquiterpene
55	τ-Cadinol	20.841	1231	-	-	1.18	-	sesquiterpene
56	a*R*-Turmerone	21.075	1240	12.34	-	-	-	sesquiterpene
57	(*Z*)-γ-Atlantone	21.136	1242	19.95	-	-	-	sesquiterpene
58	β-Turmerone	21.589	1258	14.24	-	-	-	sesquiterpene
59	α-Ketostearic acid	22.541	1293	2.57	-	-	-	acid
60	*E*-2-Tetradecen-1-ol	23.179	1318	2.88	-	-	-	alcohol
61	*Z*-1,9-Hexadecadiene	23.408	1327	1.78	-	-	-	alkadiene
62	*E*-11-Hexadecenal	23.898	1346	0.67	-	-	-	aldehyde
63	Farnesol	24.149	1355	7.29	-	-	-	sesquiterpene
	Total			99.93	99.89	99.39	99.76	

RT-retention time; RI-linear retention index.

**Table 2:** The main vibration bands from gels and dried gels.

Control	O	O Dried	AM	AM Dried	CU	CU Dried	H	H Dried	T	T Dried	Attribution
3306	3305	3296	3305	3292	3306	3296	3332	3296	3306	3296	Amide A: NH stretch coupled with a hydrogen bond
-	-	2937	-	2938	-	2933	-	2937	-	2938	Amide B: CH2 asymmetrical stretch
1638	1638	1652	1638	1657	1638	1651	1638	1656	1638	1657	Amide I: C=O stretch/hydrogen bond coupled with COO^–^
-	-	-	-	-	-	1556	-	1556	-	-	Amide II: NH bend coupled with an CN stretch
